# Mini‐Open‐Assisted Screw Fixation for Displaced Intra‐Articular Calcaneus Fractures: Three Case Reports of Patients With Good Postoperative Outcomes

**DOI:** 10.1155/cro/2414330

**Published:** 2026-01-15

**Authors:** Alexander T. M. Nguyen, Simon Males, Wagdy Ashaia

**Affiliations:** ^1^ Department of Orthopaedic Surgery, Campbelltown Hospital, Sydney, New South Wales, Australia, nsw.gov.au; ^2^ South West Sydney Clinical School, University of New South Wales, Sydney, New South Wales, Australia, unsw.edu.au; ^3^ Macarthur Clinical School, Western Sydney University, Sydney, New South Wales, Australia, westernsydney.edu.au

**Keywords:** calcaneus, case report, lower limb, percutaneous fixation, trauma

## Abstract

Displaced intra‐articular calcaneus fractures (DIACFs) are debilitating injuries often resulting from a fall from height or motor vehicle accidents. Traditional fixation methods involve extended incisions that can lead to soft tissue breakdown or necrosis. Novel techniques such as percutaneous fixation augmented by mini‐open sinus tarsi approaches are theoretically associated with fewer soft tissue complications while achieving noninferior reduction and fixation. We introduced this technique to our peripheral hospital and completed this study as part of a departmental audit and self‐critique. This descriptive case series includes three patients with DIACF who underwent fixation with the aforementioned novel technique and were followed up to observe radiological and clinical outcomes including Bohler′s angle correction, pain, return to work and quantitative scoring systems. All patients had much improved Bohler′s angle corrections (mean 35°) and were able to return to work following surgery. There were no wound issues. Our study suggests that excellent fixation of DIACFs can be achieved with a limited sinus tarsi incision and percutaneous screw fixation—without any compromise to postoperative function or soft tissue.

## 1. Introduction

Management of displaced intraarticular calcaneus fractures (DIACFs) has evolved over time. Prior to 1970, DIACFs were managed nonoperatively with immobilisation, icing and elevation followed by gradual weight bearing and physiotherapy. Patients suffered from malunion, chronic pain and difficulty weight bearing [1]. Open reduction via an extended lateral approach (LTA) or sinus tarsi approach (STA) with internal fixation was developed and was associated with improved mobility, long‐term function (> 24 months), patient satisfaction and reduced posttraumatic arthritis [2, 3]. The latest Cochrane Library Review analysed several prospective randomised clinical trials and showed that surgical management may be superior to nonsurgical management but the evidence was limited and the underpinning trials had several design flaws [1]. Furthermore, no conclusion could be drawn whether one surgical technique was better than others. With this context, development of less invasive methods has become more popular—the benefits hypothetically are reduced operating time and decreased risk of wound‐related complications. The parameters of surgical fixation for DIACFs are restoration of calcaneal length, height and angular deformity correct—the most important of which is Bohler′s angle [4]. Chongmuenwai et al.′s imaging study showed that using the screw‐only technique can reliably achieve and maintain good reduction [4]. Introduction of the mini‐open sinus tarsi–assisted calcaneus fixation to our hospital resulted in this descriptive case series as part of self‐audit. We reported on three patients with DIACFs who underwent percutaneous fixation augmented by mini‐open reduction and monitored their rehabilitation with functional scores, return to work status and radiographic parameters such as Bohler′s angle.

## 2. Methods

Our orthopaedic department is part of a 300‐bed teaching hospital that services a large area of Southwest Sydney. Common orthopaedic presentations to our hospital include distal radius, ankle and neck of femur fractures. We rarely treat high‐energy traumatic injuries as these patients are referred to the nearest tertiary hospital. However, logistical changes caused by the COVID‐19 pandemic resulted in some trauma patients being managed locally.

This descriptive case series included patients recruited consecutively between June 2022 and June 2024 who sustained DIACFs and underwent operative fixation with the method described below. We routinely conduct functional outcome scores as part of our clinical practice at follow‐up. Patients were consented to have their deidentified data, scores and imaging included in this study. Patients were excluded from the study if they declined or were unfit for operative management.

**Figure 1:**
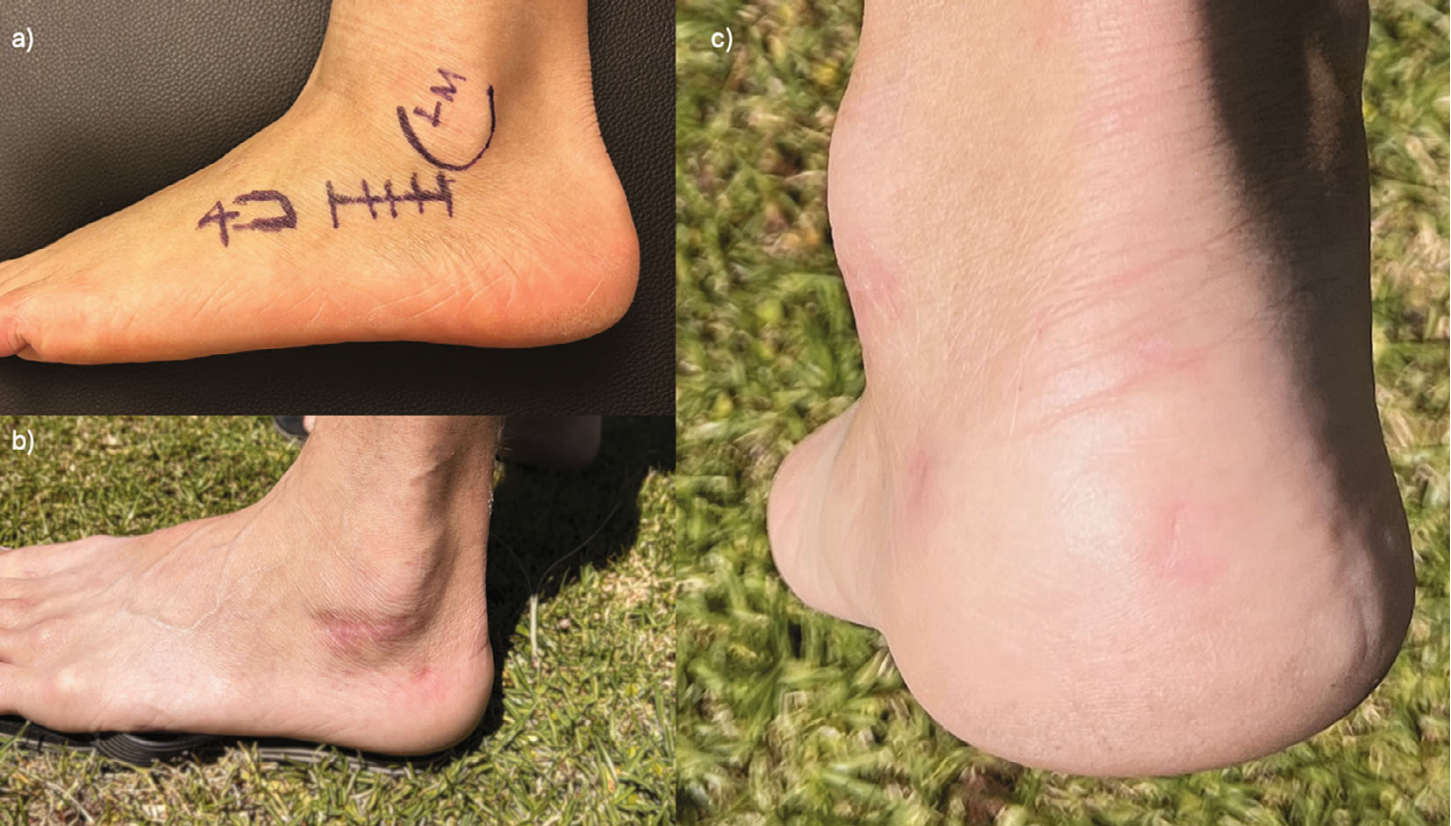
(a) Landmarks for the sinus tarsi approach. LM = lateral malleolus; 4 = 4th metatarsal base. (b, c) Healed surgical scars in a patient 1 year postsurgery and demonstrating full ability to weight bear.

All patients were reviewed by an orthopaedic registrar and the case was discussed with the senior author (W.A.). The primary surgeon for all patients was W.A. The limited sinus tarsi approach involved an incision that starts distal to the fibula and aims towards the fourth metatarsal. Percutaneous incisions were made at the posterior border of the calcaneus for screw insertion. Figure 1 demonstrates the landmarks used in this incision. Visualisation of the fracture and subtalar joint was aided by evacuation of clot. We used a combination of mini‐Houghmann retractors, periosteal, Cobb and Freers elevators to elevate depressed fragments and restore joint congruity. We used intraoperative fluoroscopy to assess the reduction and guide 1.6‐mm Kirschner wires to temporarily stabilise fracture fragments. Definitive fixation was performed with percutaneous 4‐mm screws inserted transversely to fix the subtalar joint fragments to the medial (constant) fragment while 7‐mm screws fixed the calcaneus in the postero‐anterior direction. We found that this surgical approach required much less soft tissue dissection than the larger LTA to the calcaneus, and we were still able to achieve almost anatomic reductions despite the small window. The smaller incision also allowed for easier skin closure under less tension—we used running dissolvable monofilament for the superficial skin layer. Patients were informed about potential complications from this approach including injury to the sural nerve and peroneal tendons which run close to the fibula.

Postoperatively, patients were immobilised in a short leg plaster for 1 week before switching to a walking boot to allow for early ankle range of motion exercises. Patients were nonweight bearing for 6 weeks, then upgraded to touch‐weight bearing for 2 weeks, then partial weight bearing (50% body weight) for 2 weeks, then were allowed to weight bear as tolerated in the walking boot. At 3 months, patients were advised to mobilise without a walking boot. Chenical deep venous thrombosis prophylaxis was prescribed (enoxaparin 40 mg subcutaneous injection daily for 2 months postoperatively).

Outcomes that were measured and reported included time to fixation, time to wound healing, radiological parameters including Bohler′s angle on preoperative and postoperative x‐rays and whether patients returned to work or not. Functional outcomes were assessed using Foot and Ankle Outcome Score (FAOS) and Foot Function Index (FFI). The FAOS has a range from 0 to 100 with 100 meaning no foot or ankle symptoms [5]. The FFI involves 17 questions with a score range of 0–170, with 0 meaning no foot symptoms or functional impairment [6].

## 3. Results

**Figure 2:**
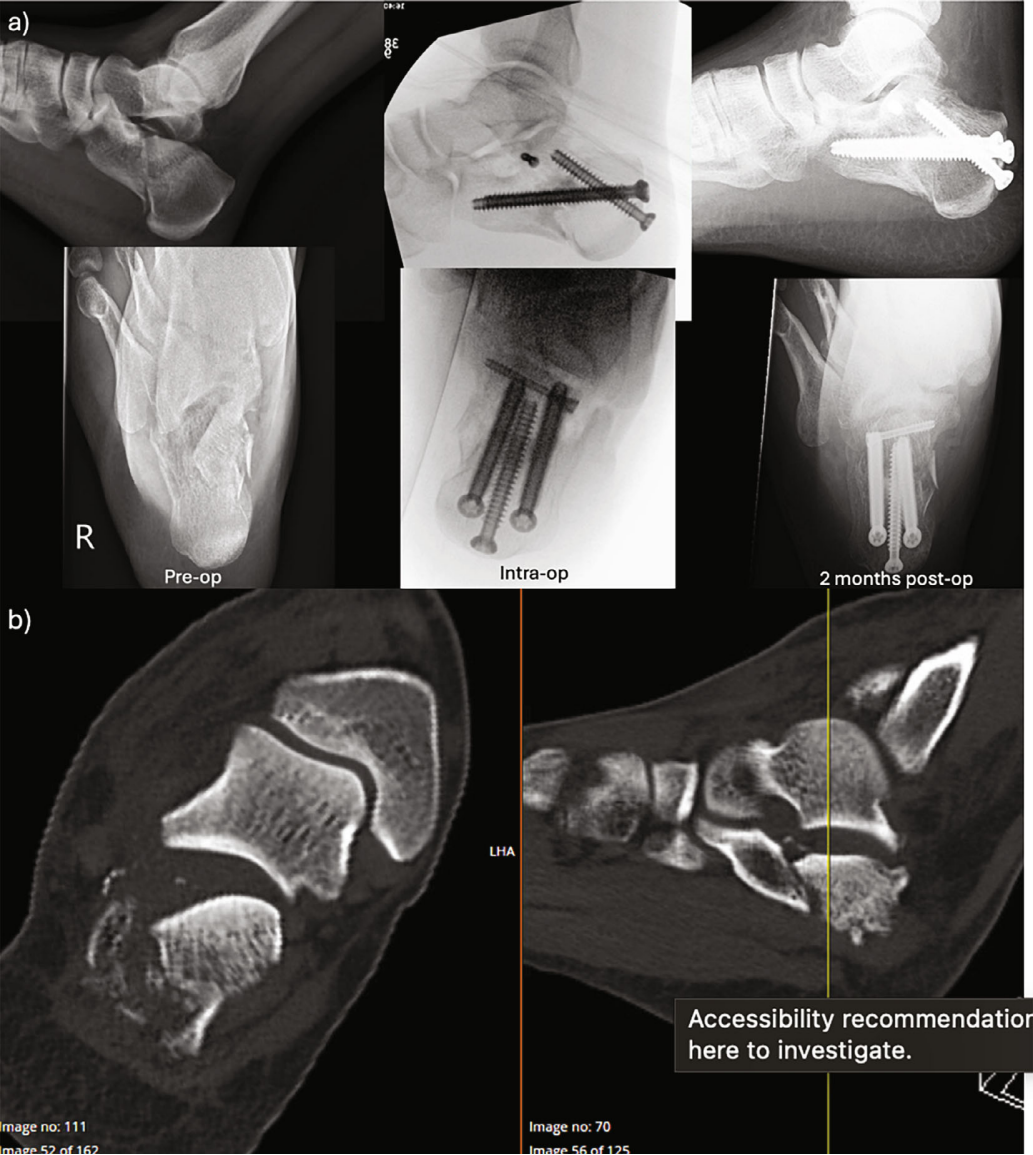
Case A. (a) Pre‐, intra‐ and postoperative x‐rays. (b) Preoperative CT scan of a closed, right‐sided Sanders 2A calcaneus fracture.

**Figure 3:**
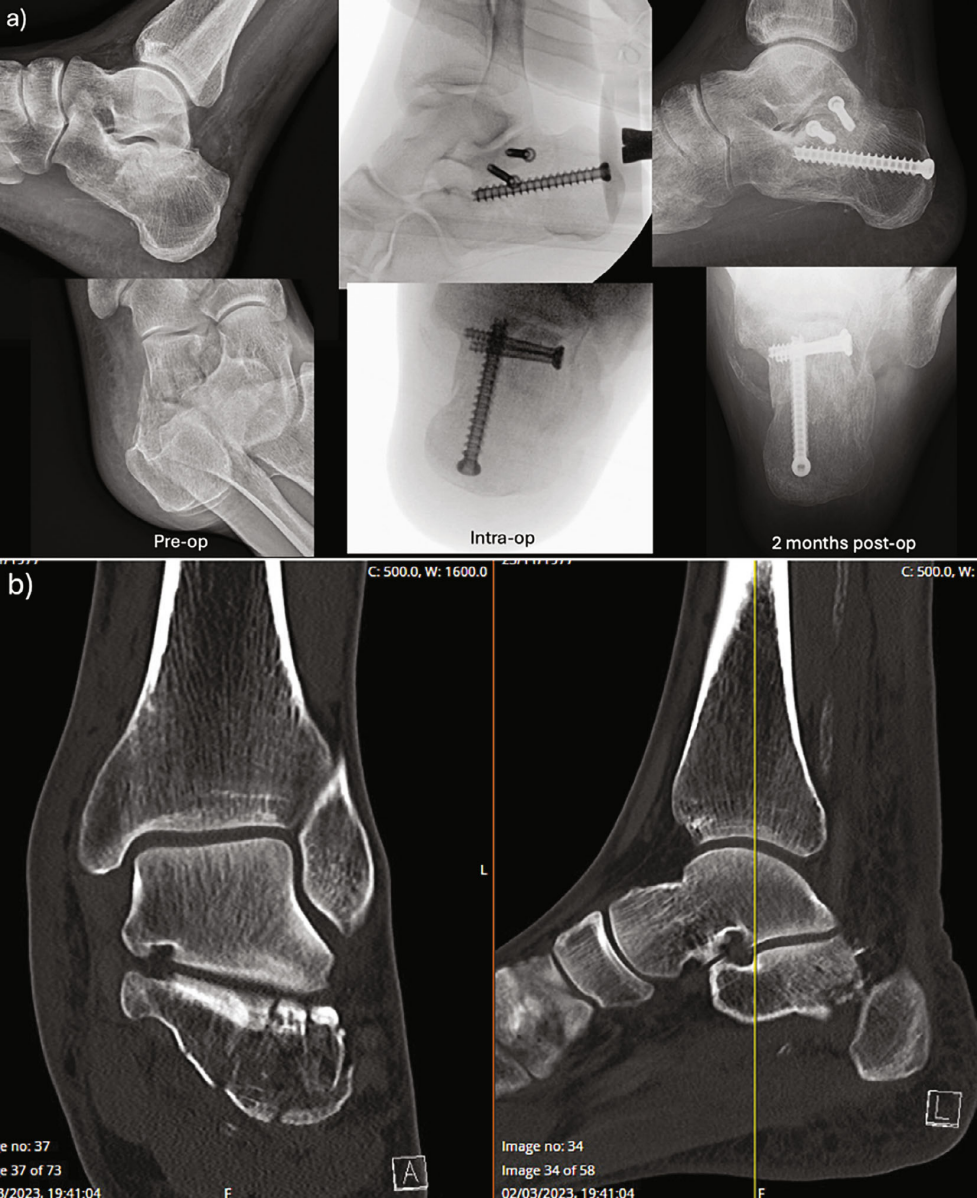
Case B. (a) Pre‐, intra‐ and postoperative x‐rays. (b) Preoperative CT scan of a closed, left‐sided Sanders 3AB calcaneus fracture.

**Figure 4:**
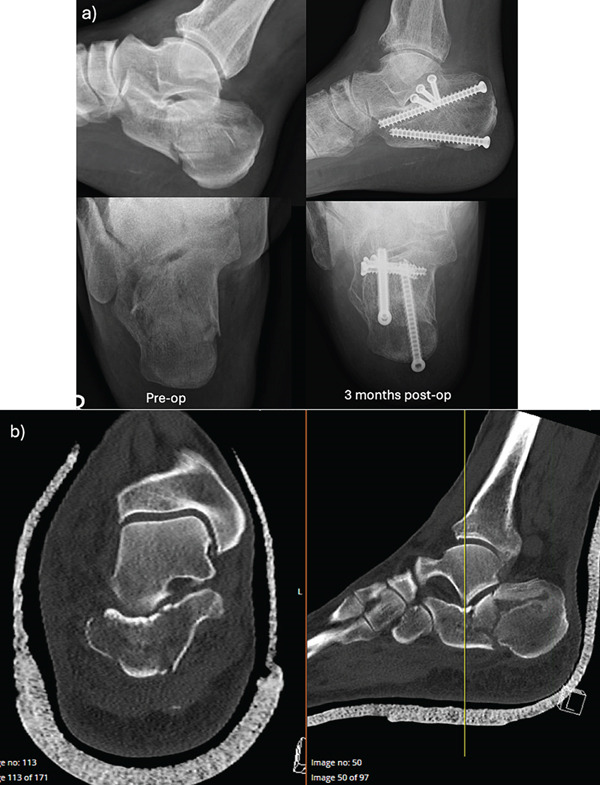
Case C. (a) Pre‐ and postoperative x‐rays. Intraoperative x‐rays unable to be retrieved. (b) Preoperative CT scan of a closed, right‐sided Sanders 2B calcaneus fracture.

Three patients were included in this study (all male, ages 43–61 years old), and all sustained their calcaneus fractures after a fall from height (Table 1 and Figures 2, 3, and 4). None of the patients had soft tissue complications, with 3 weeks being the longest time for wound healing (case C). On average, patients had an improvement in Bohler′s angle by 35° and all were able to return to work. The average FAOS and FFI scores at follow‐up were 64 and 38.3, respectively.

**Table 1:** Cases with demographics, mechanism of injury, fixation techniques and postoperative functional scores and radiological findings.

**Case**	**Age**	**Sex**	**Mechanism of injury**	**Smoking history**	**Time to fixation**	**Fixation technique**	**Post-op hospital stay**	**Pre-op Bohler**′**s angle**	**Post-op Bohler**′**s angle**	**Wound issues**	**Return to work**	**FAOS score**	**FFI score**
A	43	Male	Fall from ladder	Nonsmoker	4 days	3x percutaneous screws with sinus tarsi–assisted reduction	1 day	−10°	36°	Healed at 2 weeks post op	Yes	93 (14 months)	8
B	46	Male	Fall from ladder	Smokes 1 pack/week	5 days	3x percutaneous screws with sinus tarsi–assisted reduction	2 days	−12°	28°	Healed at 2 weeks post op	Yes	60 (29 months)	42
C	61	Male	Fall from ladder	Nonsmoker	10 days	4x percutaneous screws with sinus tarsi‐assisted reduction	1 day	2°	21°	Healed at 3 weeks post op	Yes (modified light duties)	39 (28 months)	65

## 4. Discussion

Our reported cases are in keeping with contemporary literature around less invasive fixation of calcaneus fractures. For example, the literature demonstrates a shorter operative time compared to ORIF with plates—time saved ranges from 7 to 30 min [7, 8]. Days in hospital were also shorter for those undergoing limited incision approaches (8.7 ± 3.9 vs. 17 ± 7.9 days, *p* = 0.001) [7]. Our patients had relatively short lengths of stay—most of them were discharged to wait at home for surgery, and only one patient stayed more than 1 day in our case series.

In recent published studies, the overall mean American Orthopaedic Foot and Ankle Society (AOFAS) scores at 3, 6 and final follow‐ups have been higher for less invasive fixation versus ORIF with plate [8, 9]. At the very least, there is no difference in AOFAS scores in several studies, such as one published by Zhao et al. including 728 patients [10]. All of our patients returned to work.

Increased Bohler′s angle (> 10°) was associated with better functional scores than those with < 10°—leading to increased risk of subtalar arthritis and chronic pain. Kato et al.′s study of 121 patients with Sanders Type II DIACFs showed no difference in postoperative Bohler′s angle immediately post‐op or at final follow‐up, highlighting that screw‐only fixation could maintain reduction post‐op [8]. Sato et al. showed that there may be an increased loss of Bohler′s angle correction with screw fixation compared to plate fixation, but there was no statistically significant difference in function or postoperative complications [11]. Bahaeddini et al. retrospectively reported on 187 Iranian patients; those undergoing screw fixation had less pain (visual analogue scale 3.2 vs. 4.2, *p* = 0.01), better Bohler′s angle correction (16.4° vs. 13.5°, *p* = 0.014) and lower rates of hardware removal (3.8% vs. 14.8%, *p* = 0.007) [12]. On average, we were able to correct our patient′s Bohler′s angles by 35°.

Less invasive calcaneus fixation is associated with less postoperative complications—especially wound issues—compared to traditional ORIF. Multiple studies have demonstrated this; one the largest conducted by Lappalainen et al. in 127 patients demonstrated fewer rates of deep wound infection (7.5% vs. 32%, *p* = 0.001), plastic surgery intervention (3.8% vs. 25.3%, *p* = 0.001) and wound edge necrosis (1.9% vs. 16%, *p* = 0.014) for those undergoing less invasive fixation compared to ORIF [13]. In our case series, none of the patients suffered from complications post‐op at the time of writing.

The limitations of this study primarily stem from the study design. As a descriptive case series, the small sample size and lack of control or comparison group mean that our results may not accurately reflect the broader community (sampling bias). This is hard to rectify in our hospital due to the very small number of patients presenting with surgically amenable DIACFs (three patients in 2 years). Future studies to produce higher quality evidence require a larger sample size and control group that is adequately powered to detect meaningful differences in outcomes—in our community this will require cooperation between multiple hospitals.

## 5. Conclusion

Calcaneus fractures are serious injuries with potential for significant morbidity from foot deformity, chronic pain and posttraumatic arthritis. Although undisplaced, extra‐articular fractures have been historically well managed with conservative methods, displaced and intraarticular fracture patterns are a topic of ongoing discussion. Novel techniques such as percutaneous screw fixation with mini sinus tarsi–assisted reduction are associated with less soft tissue complications while allowing for noninferior reduction and fracture fixation. The outcomes of our three patients were in line with contemporary literature—all were able to return to work and none had any soft tissue complications.

## Ethics Statement

Approval was provided by Quality Manager ME (mary.edappulavan@health.nsw.gov.au) at Campbelltown Hospital in accordance with Section 2e of the National Health and Medical Research Council “Ethical Considerations in Quality Assurance and Evaluation Activities.” This is Quality Improvement Project CT19‐2024.

## Consent

Patients provided written consent for their deidentified information to be used in our academic article.

## Conflicts of Interest

The authors declare no conflicts of interest.

## Funding

No funding was received for this research.

## Data Availability

The data that support the findings of this study are available on request from the corresponding author. The data are not publicly available due to privacy or ethical restrictions.
